# Cellular NADH and NADPH Conformation as a Real-Time Fluorescence-Based Metabolic Indicator under Pressurized Conditions

**DOI:** 10.3390/molecules26165020

**Published:** 2021-08-19

**Authors:** Martin Heidelman, Bibek Dhakal, Millicent Gikunda, Kalinga Pavan Thushara Silva, Laxmi Risal, Andrew I. Rodriguez, Fumiyoshi Abe, Paul Urayama

**Affiliations:** 1 Department of Physics, Miami University, Oxford, OH 45056, USA; mheidelm@nd.edu (M.H.); bibek.dhakal@vanderbilt.edu (B.D.); mngikund@uark.edu (M.G.); silvap@miamioh.edu (K.P.T.S.); Laxmi.Risal@utdallas.edu (L.R.); rodrigai@miamioh.edu (A.I.R.); 2 Department of Chemistry and Biological Science, College of Science and Engineering, Aoyama Gakuin University, Sagamihara 252-5258, Japan; abef@chem.aoyama.ac.jp

**Keywords:** yeast, hydrostatic pressure, autofluorescence, spectral phasor analysis, NADH and NADPH conformation

## Abstract

Cellular conformation of reduced pyridine nucleotides NADH and NADPH sensed using autofluorescence spectroscopy is presented as a real-time metabolic indicator under pressurized conditions. The approach provides information on the role of pressure in energy metabolism and antioxidant defense with applications in agriculture and food technologies. Here, we use spectral phasor analysis on UV-excited autofluorescence from *Saccharomyces cerevisiae* (baker’s yeast) to assess the involvement of one or multiple NADH- or NADPH-linked pathways based on the presence of two-component spectral behavior during a metabolic response. To demonstrate metabolic monitoring under pressure, we first present the autofluorescence response to cyanide (a respiratory inhibitor) at 32 MPa. Although ambient and high-pressure responses remain similar, pressure itself also induces a response that is consistent with a change in cellular redox state and ROS production. Next, as an example of an autofluorescence response altered by pressurization, we investigate the response to ethanol at ambient, 12 MPa, and 30 MPa pressure. Ethanol (another respiratory inhibitor) and cyanide induce similar responses at ambient pressure. The onset of non-two-component spectral behavior upon pressurization suggests a change in the mechanism of ethanol action. Overall, results point to new avenues of investigation in piezophysiology by providing a way of visualizing metabolism and mitochondrial function under pressurized conditions.

## 1. Introduction

Reduced pyridine nucleotides (e.g., reduced nicotinamide adenine dinucleotide (NADH) and nicotinamide adenine dinucleotide phosphate (NADPH)) are metabolic cofactors known for their role in energy metabolism and antioxidant defense, respectively, along with involvement in calcium homeostasis, gene expression, immunological functions, aging, and cell death [1,2]. Excited-state emission from NADH and NADPH is the primary component of UV-excited cellular autofluorescence (endogenous fluorescence) and is widely used in biotechnology and biomedicine [3] (The abbreviation NAD(P)H is often used to denote the autofluorescence signal originating from both NADH and NADPH, since they cannot be discriminated due to their nearly identical fluorescence spectral properties [4]). Here, we demonstrate the use of NADH and NADPH conformation sensed from UV-excited autofluorescence as a real-time metabolic indicator under pressurized conditions.

Generally, cellular processes associated with biological membranes and multimeric associations exhibit pressure sensitivity; e.g., membrane protein function is disrupted at 25–50 MPa (0.101 MPa = 1 atm) and ribosomal dissociation begins at 60 MPa as compared with the 200 or so MPa pressure needed for monomeric protein denaturation [5,6,7,8]. Regarding respiration, pressure-regulated respiratory oxidases and cytochromes are found in piezophilic microbes *Shewanella benthica* [9,10], *Sh. violacea* [11,12], and *Photobacterium profundum* [13,14]. Pressure affects cellular respiratory activity in eukaryotes as well, reducing oxygen consumption rates [15]. The presence of piezotolerant obligate aerobic yeasts in deep sea environments further justifies investigating pressure effects on respiratory mechanisms [16,17].

Information on NADH- and NADPH-linked metabolism under pressure also has applications in agriculture and food technologies. For example, real-time monitoring of NADH may be useful in bioethanol production, which is influenced by pressure [18] or in improving the flavor and quality of brewed products because high NADH availability is a factor for maintaining low acetaldehyde content during alcoholic fermentation [19,20]. With the usefulness of pressure as a biophysical tool being well recognized [8,21,22,23], extending techniques for the real-time monitoring of cellular NADH and NADPH conformation to pressurized conditions provides a label-free approach for investigating a range of NADH- and NADPH-linked metabolic function.

Ambient pressure analysis of autofluorescence signals has identified multiple cellular NADH and NADPH conformations, which is significant because the distribution of conformational forms depends on metabolic conditions [24,25,26]. For example, it is the free (as opposed to protein-bound) cellular NADH pool that is shared by the various NADH-related dehydrogenases, and that is the determinant of reaction velocities [27]. The ability to sense conformation beyond a “free *versus* protein bound” description suggests that detailed metabolic information resides in autofluorescence signals, leading to a renewed interest in developing NADH and NADPH conformation as a metabolic indicator and endogenous biomarker at ambient pressure [3,4,24,25,26,28,29].

Since conformation affects the emission spectrum [30], spectrum shape is a source for contrast in sensing cellular metabolic response [31]. Spectral phasor analysis applied to UV-excited autofluorescence from cellular suspensions can distinguish between metabolic transitions involving multiple NADH- and NADPH-utilizing pathways due to its ability to test for two-component behavior in the spectral response [32,33,34].

Here, we use UV-excited autofluorescence spectroscopy to sense cellular NADH and NADPH conformation during real-time metabolic monitoring of cellular samples under pressurized conditions. Using *Saccharomyces cerevisiae* (baker’s yeast) as a model organism for piezophysiology [6], we first demonstrate real-time metabolic monitoring under pressurized conditions by comparing the cyanide-induced autofluorescence response at ambient and 32 MPa pressure. Similarities in the response suggest cyanide’s mechanism of respiratory inhibition is not significantly impaired at this pressure. Interestingly, a change in pressure itself also induces a change in the autofluorescence intensity without a significant change in spectrum shape. We use pressure cycling (up to 32 MPa, 30 min period) to explore this pressure-induced response, finding that increasing pressure reduces autofluorescence intensity and vice versa. The change in spectrum shape between subsequent pressurizations does not follow two-component spectral behavior, suggesting a persistence in the pressurization response that has a metabolic component, since it does not follow the piezochromic response of NADH in solution. Based on these observations, we propose a pressure-induced change in cellular redox state and reactive oxygen species (ROS) production. Finally, we present the autofluorescence response to ethanol as an example of one that is altered under pressurized conditions. We find that while the ethanol-induced response follows two-component spectral behavior at ambient pressure, the response develops non-two-component behavior under pressure, suggesting the involvement of multiple mechanisms over the response duration and indicating the presence of pressure-dependent dynamics for NADH- and NADPH-linked metabolisms.

## 2. Results

### 2.1. Cellular Autofluorescence Response to Cyanide

**Figure 1:**
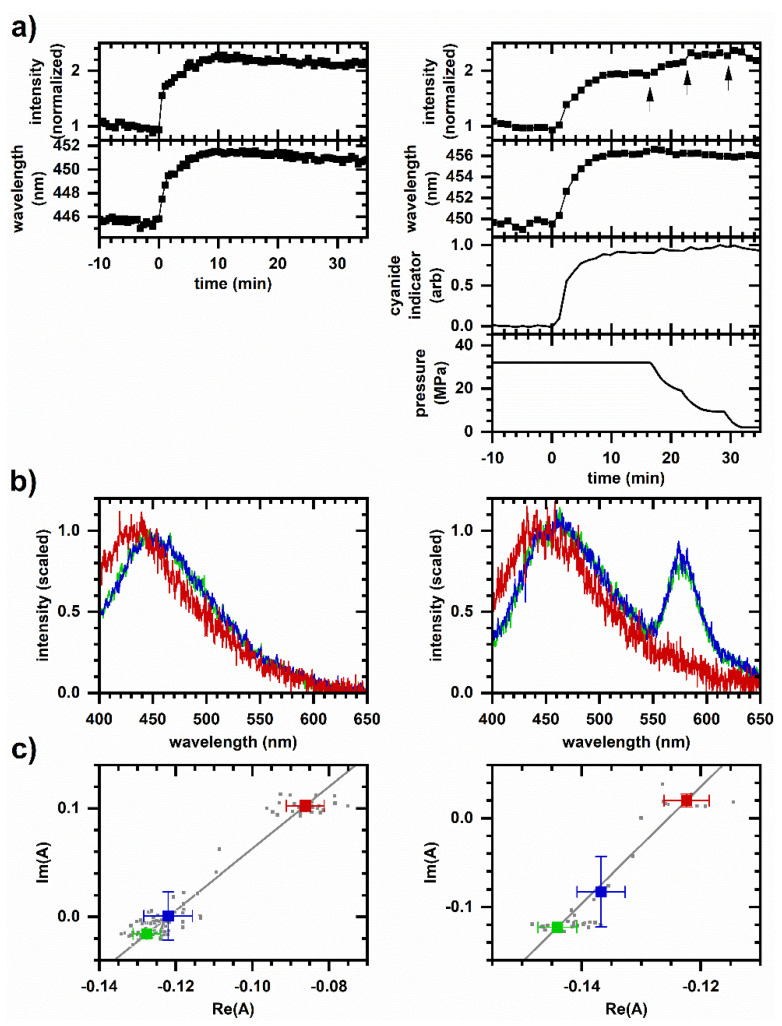
Cellular autofluorescence response to cyanide. Left column, 8 mM cyanide at ambient pressure in a spectroscopic cuvette. Right column, 25 mM cyanide at 32 MPa pressure in the microperfusion system. Representative responses are shown; behavior is reproduced on *N* independently prepared samples, *N* > 50 at ambient pressure and *N* = 4 at high pressure. (**a**) Autofluorescence intensity and average emission wavelength versus time. Spectrally integrated autofluorescence intensity is normalized to the intensity averaged over 10 min prior to cyanide introduction. Autofluorescence wavelength is plotted as an intensity-weighted average emission wavelength. Time is shifted so that cyanide is introduced to the sample at *t* = 0 min. For the microperfusion data, 1 µM rhodamine is added to the reservoir as a cyanide indicator; shown is the integrated emission intensity for pixels 675–725 (572–585 nm wavelength) scaled to be between 0 and 1. The measured pressure is also shown. Arrows indicate intensity increases when pressure is released. (**b**) Representative emission spectra. Red—Prior to cyanide introduction; blue—10 min after cyanide introduction; green—20 min after cyanide introduction. Spectra are scaled to minimize the least-squares difference. For the microperfusion data, the peak at 575 nm wavelength is due to the rhodamine indicator. (**c**) Phasor plots. Small symbols are phasors calculated from individual measurements. Large symbols are average and standard deviations of phasor values. Color corresponds to time intervals over which phasor values are averaged: red—10 min prior to cyanide introduction; blue—first 10 min after cyanide introduction; green—second 10 min after cyanide introduction. Gray line is a linear least-squares fit to the large symbols. Intensity, average emission wavelength, phasor, and least-square minimization are calculated from a measured spectrum using the first 400 pixels (400–500 nm wavelength).

As a demonstration of real-time metabolic monitoring under pressurized conditions, we compare the UV-excited autofluorescence response to cyanide at ambient and 32 MPa pressures (Figure 1). The responses are similar with an increase in the emission intensity and a shift to longer emission wavelength after cyanide introduction (Figure 1a,b). Spectral phasors (Figure 1c) also show a shift consistent with an increasing emission wavelength. For each case, phasor values averaged over 10-min intervals prior to and after cyanide introduction share a collinear relationship, indicating that the autofluorescence response follows two-component behavior over time and suggesting that a single mechanism causes the spectral change over the duration of the response. Interestingly, as pressure is decreased, the emission intensity again increases (as indicated by the arrows in Figure 1a), although this time without significant change in the emission wavelength.

### 2.2. Cellular Autofluorescence Response to Pressure Cycling

**Figure 2:**
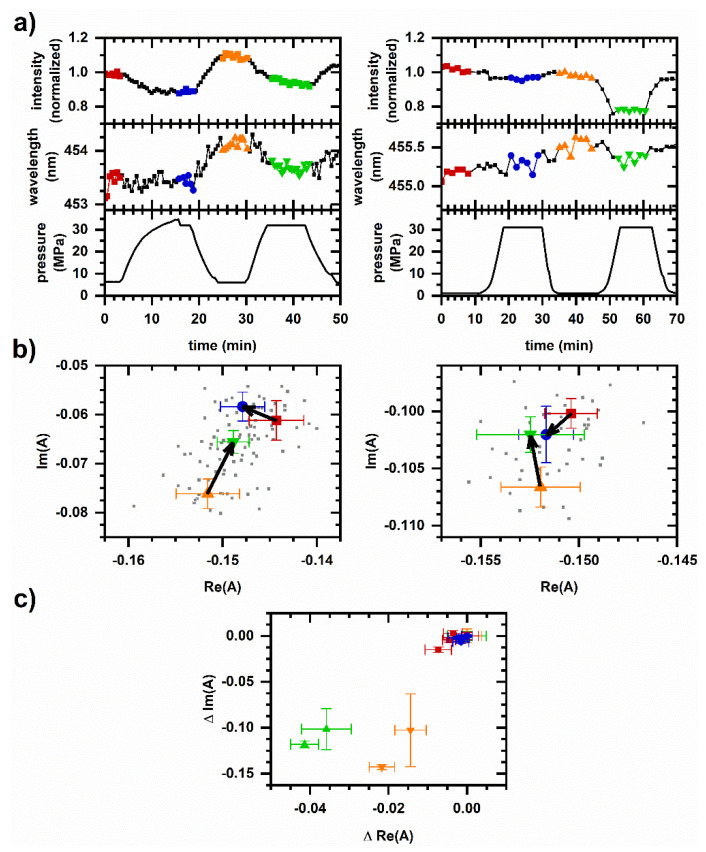
Cellular autofluorescence response to pressure cycling. Representative results are shown; each column features data from an independently prepared sample. Behavior is reproduced on *N* = 5 independently prepared samples. (**a**) Autofluorescence intensity and average emission wavelength versus time. Spectrally integrated autofluorescence intensity is normalized to the average intensity prior to the initial pressurization. An autofluorescence wavelength is calculated as an intensity-weighted average emission wavelength. The measured pressure is also shown. (**b**) Phasor plots. Small symbols are phasors calculated from individual measurements. Large symbols are average and standard deviations of phasor values. Color corresponds to time intervals of constant pressure over which phasor values are averaged; the corresponding data points are shown in the same color in Figure 2a. Arrows indicate phasor shift directions upon pressurization. The intensity, average emission wavelength, and phasor are calculated from a measured spectrum using the first 400 pixels (400–500 nm wavelength). (**c**) Comparison of phasor plots for cyanide and pressure cycling data. Phasor values are shifted so that the initial phasor is at the origin. The datasets are red—Pressure cycling, Figure 2b, left; blue—Pressure cycling, Figure 2b, right; green—Cyanide response at ambient pressure, Figure 1c, left; and orange—Cyanide response at 32 MPa, Figure 1c, right.

To gain insight as to whether this pressure-induced intensity increase is of metabolic origin or due to piezochromic effects, we note that comparable changes in emission intensity (10–20%) along with a correspondingly small change to the autofluorescence spectrum (less than a nanometer change in the average emission wavelength) occurs during pressure cycling even without the introduction of chemicals. Figure 2a shows the autofluorescence intensity during cycling between ambient or near-ambient pressure and 32 MPa pressure with a period of approximately 30 min. The emission intensity increases with depressurization as in Figure 1a; conversely, intensity decreases upon pressurization. Figure 2b shows the phasor response during pressure cycling. Phasors shift in a negative *Re*(*A*) direction during the first pressurization, a negative *Im*(*A*) direction during the first depressurization, then a positive *Im*(*A*) direction during the second pressurization. Overall, spectral phasors show non-collinear shifts between pressurization and depressurization and between subsequent pressurization cycles, suggesting multiple mechanisms for spectral change are at play. Although small compared with the response to cyanide (Figure 2c), there is a reproducible structure in the phasor response during cycling.

### 2.3. Excited-State Emission from NADH in Solution under Pressurized Conditions

**Figure 3:**
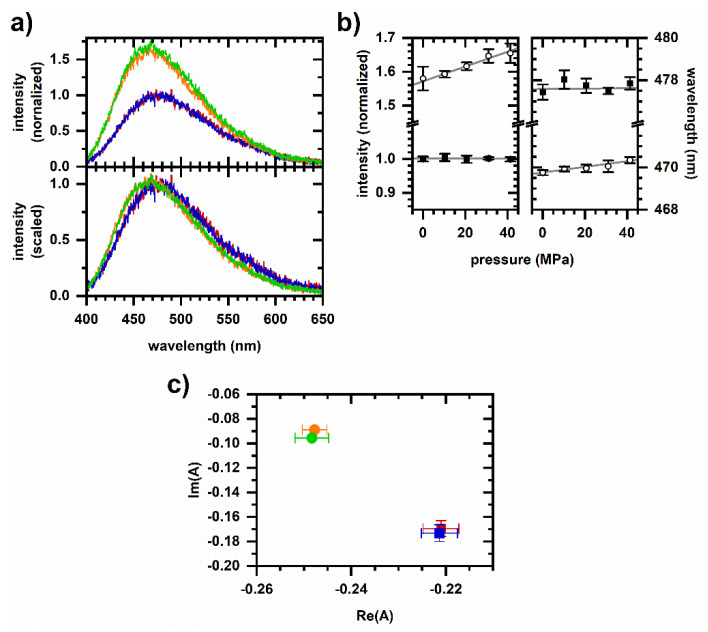
Excited-state emission from NADH in solution under pressurized conditions. (**a**) Emission spectra for 40 µM NADH in 0 vol% methanol (red, 0.1 MPa; blue, 41.4 MPa) and 90 vol% methanol (orange, 0.1 MPa; green, 41.4 MPa). Top, spectra are normalized to the peak intensity of the 0 vol% methanol, ambient pressure spectrum. Bottom, spectra are scaled to minimize least-squares differences. (**b**) Peak intensity and peak-intensity wavelength of emission from 0 vol% methanol (solid square) and 90 vol% methanol (open circle) samples. Values are from a Gaussian fit to a 75 nm wavelength interval roughly centered on the spectrum peak. Error bars are the standard deviation of repeated measurements which include spectra taken during both the pressurization and depressurization legs of a data run. Least-squares linear fits are shown in gray. (**c**) Phasor plot. Symbol color corresponds to the same conditions as in Figure 3a. Phasors are calculated over the first 512 pixels (400–530 nm wavelength).

We compare pressure cycling results with the piezochromic response of NADH solutions. Figure 3 shows spectra from NADH in solutions of varying polarity; methanol (from 0 to 90 vol%) instead of ethanol is used to vary the polarity due to the availability of previous studies on excited-state dynamics in water–methanol mixtures, which indicate an opening of the molecular conformation with increasing methanol [35,36,37]. We observe an increase in intensity and a shift to shorter emission wavelength as the methanol concentration is increased. As a given sample is pressurized, there is an increase in emission intensity and a (slight) shift to a longer wavelength with these effects being greater at higher methanol concentrations. Piezochromic effects on NADH emission at these pressures are small compared with solvatochromic effects.

Note that piezochromic effects (Figure 3) cannot account for the cellular autofluorescence response to pressure cycling (Figure 2). Pressurization increases the emission intensity in solution, while it decreases the autofluorescence intensity. Pressurization increases the emission wavelength for NADH in solution, while it has a small effect on the autofluorescence emission wavelength during the first pressurization and decreases the wavelength during the second pressurization.

**Figure 4:**
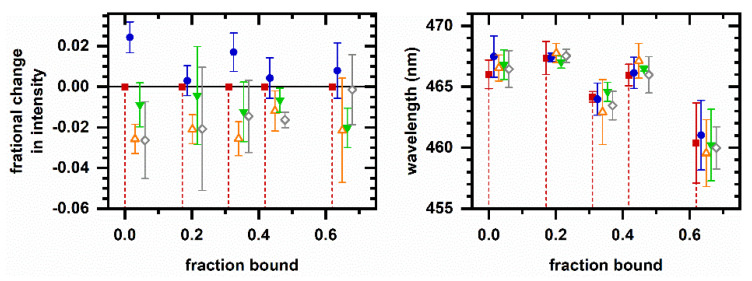
Excited-state emission from NADH and protein solutions during pressure cycling. For both plots, the estimated fraction of NADH bound to MDH is indicated by the drop line; data points after subsequent pressurization and depressurization are offset to the right for clarity. NADH concentration is 5 µM prior to MDH addition. Color/symbol indicates condition: red/square—Prior to pressurization; blue/circle—After first pressurization; orange/upright triangle—After first depressurization; green/inverted triangle—After second pressurization; and gray/diamond—After second depressurization. Filled symbols are pressurizations to 33.1 MPa; open symbols are depressurizations to 1.4 MPa. **Left**, fractional change in emission intensity after changing pressure. **Right**, intensity-weighted average emission wavelength. Error bars are standard deviations of at least five measurements.

Effects of pressure cycling on protein-bound NADH are shown in Figure 4. The uncertainties are larger here than in Figure 3 because a smaller NADH concentration was used to cover a range in the fraction bound. The initial pressurization and depressurization of the solution increases and then decreases the emission intensity, respectively, although the response to subsequent pressure cycling is variable. These responses are again opposite to those observed in the cellular autofluorescence, and changes in intensity are small (less than 3%) as compared with changes in autofluorescence (10–20%). Finally, piezochromic effects on emission wavelength remain small even in the sample having a large protein bound fraction. Together, observed piezochromic effects (Figure 3 and Figure 4) do not account for the response of cellular autofluorescence to pressure cycling (Figure 2).

### 2.4. Cellular Autofluorescence Response to Ethanol

**Figure 5:**
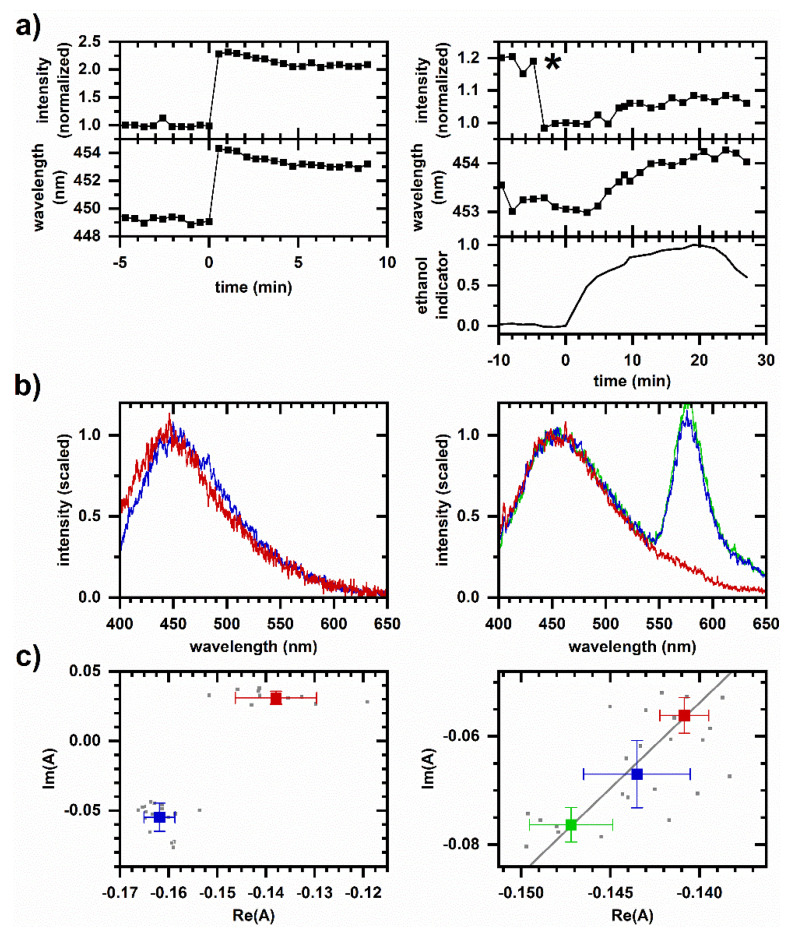
Cellular autofluorescence response to ethanol at ambient pressure. Left column, 2 vol% ethanol, in spectroscopic cuvette. Right column, 10 vol% ethanol, in a microperfusion system. Representative responses are shown; behavior is reproduced on *N* independently prepared samples, *N* > 50 in a cuvette and *N* = 3 in the microperfusion system. (**a**) Autofluorescence intensity and average emission wavelength versus time. Spectrally integrated autofluorescence intensity is normalized to the intensity averaged over 10 min prior to ethanol introduction. Autofluorescence wavelength is calculated as an intensity-weighted average emission wavelength. Time is shifted so that ethanol is introduced to the sample at *t* = 0 min. For the microperfusion data, 1 µM rhodamine is added to the reservoir as an ethanol indicator; shown is the integrated emission intensity for pixels 675–725 (572–585 nm wavelength) scaled to be between 0 and 1. The sudden drop in intensity at (*) is an artifact of sample movement within the capillary chamber. (**b**) Representative emission spectra. Red—Prior to ethanol introduction; blue—10 min after ethanol introduction; green—20 min after ethanol introduction. Spectra are scaled to minimize least-square difference. For the cuvette data, a 20 min spectrum (green) is not shown because the emission reached a steady value within the first 5 min. For the microperfusion data, the peak at 575 nm wavelength is due to the rhodamine indicator. (**c**) Phasor plots. Small symbols are phasors calculated from individual measurements. Large symbols are average and standard deviations of phasor values. Color corresponds to time intervals over which phasor values are averaged: red—10 min prior to ethanol introduction; blue—First 10 min after ethanol introduction; green—Second 10 min after ethanol introduction. The gray line is a linear least-squares fit to the large symbols. Intensity, average emission wavelength, phasor, and least-square minimization are calculated from a measured spectrum using the first 400 pixels (400–500 nm wavelength).

Finally, in contrast to the cyanide-induced autofluorescence response (Figure 1), we present a chemically induced autofluorescence response that is significantly altered by pressurization. As a positive control, Figure 5 shows the autofluorescence response to ethanol at ambient pressure both in a spectroscopic cuvette and using the microperfusion system. Although the response is smaller and slower in the microperfusion system possibly due to the slower rise in ethanol concentration as the fluid reservoirs are switched, the two responses are similar. Ethanol induces an increase in emission intensity and a shift to longer emission wavelength. The phasor response appears to follow two-component behavior over the duration of the response; i.e., the phasor values averaged over subsequent 10 min intervals prior to and after ethanol introduction share a collinear relationship.

**Figure 6:**
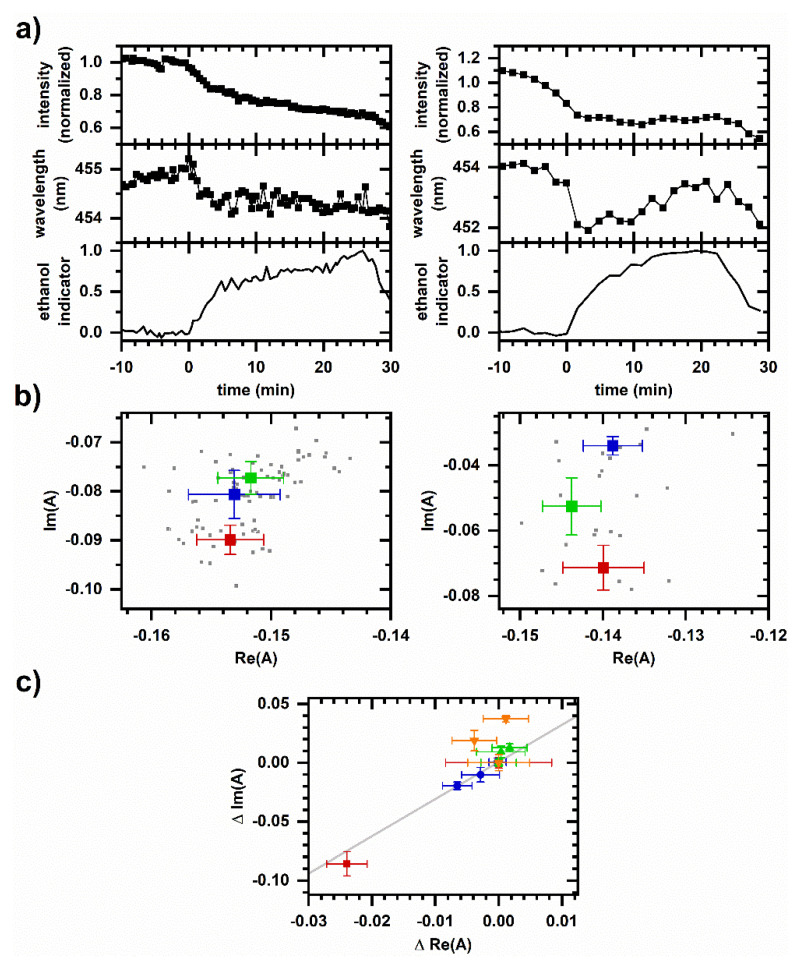
Cellular autofluorescence response to ethanol under pressurized conditions. *Left column*, 5 vol% ethanol, 12 MPa. *Right column*, 5 vol% ethanol, 30 MPa. Representative responses are shown; behavior is reproduced on *N* independently prepared samples, *N* = 3 at each pressure. (**a**) Autofluorescence intensity and average emission wavelength versus time. Spectrally integrated autofluorescence intensity is normalized to the intensity averaged over 10 min prior to ethanol introduction. Autofluorescence wavelength is calculated as an intensity-weighted average emission wavelength. Time is shifted so that ethanol is introduced to the sample at *t* = 0 min. 1 µM rhodamine is added to the reservoir as an ethanol indicator; shown is the integrated emission intensity for pixels 675–725 (572–585 nm wavelength) scaled to be between 0 and 1. (**b**) Phasor plots. Small symbols are phasors calculated from individual measurements. Large symbols are average and standard deviations of phasor values. Color corresponds to time intervals over which phasor values are averaged: red—10 min prior to ethanol introduction; blue—First 10 min after ethanol introduction; green—Second 10 min after ethanol introduction. Intensity, average emission wavelength, and phasor are calculated from a measured spectrum using the first 400 pixels (400–500 nm wavelength). (**c**) Comparison of phasor plots for ethanol-induced response data. Phasor values are shifted so that the initial phasor is at the origin. The datasets are red/square—ambient pressure, Figure 5c, left; blue/circle—Ambient pressure in perfusion system, Figure 5c, right; green/upright triangle—Response at 12 MPa, Figure 6b, left; and orange/inverted triangle—Response at 30 MPa, Figure 6b, right. Gray line is a linear least-squares fit to ambient pressure, microperfusion data (blue circles) only.

Figure 6 shows the autofluorescence response to ethanol under pressurized conditions (12 MPa and 30 MPa pressure). There are decreases in emission intensity and emission wavelength corresponding to the arrival of ethanol, as opposed to increases in intensity and wavelength observed at ambient pressure. Notably, spectral phasors indicate possible non-two-component behavior over the duration of the response, suggesting a response that has a time-dependent mechanism, i.e., because the average spectrum from 10 to 20 min after ethanol arrival is not a linear superposition of the unperturbed spectrum and the average spectrum from 0 to 10 min after ethanol arrival; the mechanism for spectral response may have changed.

Figure 6c shows phasors for both the ambient and high-pressure responses on the same plot. Ambient pressure responses show mutual two-component behavior, while the spectral responses at high pressure show significant deviation from the linear fit line.

## 3. Discussion

First, we relate cellular autofluorescence to conformation by considering NADH emission properties [30]. Since the oxidized form is not fluorescent, an increase in emission intensity indicates an increase in concentration or an increase in quantum efficiency of the reduced form. Concentration increases when cellular redox shifts to a more reduced state or when the total (i.e., combined oxidized and reduced) concentration increases. Quantum efficiency increases with protein binding, but this is often accompanied by a decrease in emission wavelength.

For example, ethanol and cyanide are both oxidation inhibitors at ambient pressure [38,39,40], and so the autofluorescence intensity increases when ethanol or cyanide is introduced and the cellular system shifts toward reduction [41]. There is also a shift to longer emission wavelength associated with an increased proportion of free (as opposed to protein bound) NADH. Detailed analysis of autofluorescence spectrum shape, e.g., using phasor analysis, reveals that the spectral responses associated with ethanol and cyanide can be distinguished [32].

Here, UV-excited autofluorescence provides real-time information on the cellular metabolic response under pressurized conditions. Although the overall response to cyanide is similar between ambient and 32 MPa pressure (Figure 1), an unexpected behavior is the increase in autofluorescence intensity upon depressurization (Figure 1, arrows). Characterized further using pressure cycling (Figure 2), depressurization is also accompanied by a small emission wavelength increase. Since piezochromic effects (Figure 3) do not account for the increase in intensity, there is either an increase in NADH or NADPH concentration and/or increase in protein-bound proportion. However, an increase in the protein-bound proportion would result in a shift to shorter emission wavelength. Since there is a shift toward longer wavelengths, there is not an increase in the protein-bound proportion. The reason for the intensity increase appears to be an increase in concentration upon depressurization, which is likely due to a shift in redox state toward reduction rather than an overall increase in total concentration due to the rapidness of the intensity change.

Note that the cellular autofluorescence response due to the second pressurization, i.e., 10–20% decrease in emission intensity and shift to shorter emission wavelength (Figure 2), is similar to the autofluorescence response to peroxide [34], and so this change in redox state may involve pressure-induced ROS production. This is consistent with previous observations of pressure-induced increase in ROS concentrations and oxidative stress, e.g., in *S. cerevisiae* pressurized to between 25 and 50 MPa for 30 min [42] and in *Escherichia coli* at 150–400 MPa [43]. A connection between redox and oxidative stress is possible given that NADH-dependent ROS generation from mitochondria and ROS generation from NADPH oxidases are two key sources of cellular ROS [1,2]. If the mechanism for the proposed pressure-induced shift to reduction involves a differential pressure-induced modulation of oxidative and/or reductive pathways, note that the autofluorescence response to cyanide (an oxidation inhibitor) did not appear to be significantly affected (Figure 1).

Next, Figure 5 and Figure 6 illustrate sensing of a cellular autofluorescence response altered by pressurization. When ethanol is introduced at ambient pressure, the autofluorescence intensity increases and shifts to longer emission wavelength. Under pressurized conditions (12 and 30 MPa), the opposite behavior is observed, suggesting a shift toward oxidation and an increased protein-bound proportion.

Although both ethanol and cyanide are oxidation inhibitors at ambient pressure, the autofluorescence response to ethanol exhibits greater pressure sensitivity, presumably indicating a pressure sensitivity in the mechanism of ethanol action. Cyanide acts through binding to Complex IV of the electron transport chain [40], while ethanol is believed to have a less specific mechanism involving biological membranes [38,39]. Generally, functions associated with biological membranes are known to be pressure sensitive [6,8], and so the greater pressure sensitivity of the ethanol response is reasonable. We do not yet have a model for this pressure sensitivity, although we note that pressures here are milder than the 50 MPa used to investigate high-pressure activation of stress responses [44]. The autofluorescence response in Figure 6 is again similar to the autofluorescence response to peroxide [34], which is consistent with ethanol being a source for oxidative stress in yeast [45].

The use of phasor analysis and the assessment of two-component spectral behavior in the autofluorescence response suggest new avenues for investigating pressure-induced phenomena. For example, ethanol-induced autofluorescence response follows two-component behavior over the duration of the response at ambient pressure (Figure 5) but not at high pressure (Figure 6). Previously, we tested the interpretation that two-component behavior in the autofluorescence response occurs when sequentially induced metabolic change involves the same response mechanism and non-two-component behavior can occur when metabolic change involves different response mechanisms using a range of chemicals known to affect glycolysis, mitochondrial function, and oxidative stress [33,34]; e.g., the response to sequential additions of ethanol and cyanide showed non-two-component behaviors, and so the two responses were distinguishable. Therefore, the non-two-component behavior in Figure 6b suggests the mechanism for ethanol-induced autofluorescence response under pressure changes over the response duration. 

The non-collinear phasor shifts between pressurization and depressurization and between subsequent pressurization cycles (Figure 2) may be another example where the mechanism for autofluorescence response is dependent on time. Past studies observing yeast budding as an indicator for stress have suggested that metabolic change continues to occur even after release from a 30 min exposure to 50 MPa pressure [46]. Since membrane-associated systems tend to be pressure sensitive, one source for observed pressure cycling effects may be the disruption or regulation of the mitochondrial respirasome. In *S. cerevisiae*, the respirasome is believed to be a supercomplex comprised of Complexes III and IV with a loosely associated NADH dehydrogenase [47].

Note that “two-component spectral behavior” does not imply that emission is comprised of two spectral components. In fact, UV-excited autofluorescence is comprised of emission from other endogenous fluorophores [3] in addition to the many possible conformations of cellular NADH and NADPH. Spectral components may be identifiable using singular value decomposition or similar approach. Here, we are performing bulk fluorescence measurements, and so a “component” is understood in terms of the ensemble emission from the cellular sample. “Two-component spectral behavior” means the autofluorescence spectrum is being described as emission from a superposition of two conformational ensembles; e.g., it is associated with the activated and inactivated forms of a metabolic pathway. Non-two-component behavior means more than two ensembles are needed to model an autofluorescence response, suggesting the involvement of multiple pathways or metabolic response mechanisms. Previous measurements demonstrating this behavior are described in Section 4.3, and additional discussion is found in previous studies [32,33,34].

Finally, we reflect on the broader significance of the high-pressure autofluorescence studies shown here. Deep-ocean pressures can exceed 100 MPa, and so the pressures used here are relevant to life [5,6,7,8]. Although a change of tens of MPa pressure is not a typical physiological range for *S. cerevisiae*, it may encounter these changes during food or biotechnological processing [18]. On the other hand, these pressure changes may be physiological for organisms inhabiting high-pressure environments, and so *S. cerevisiae* serves as a model system for piezophysiology [6].

The metabolic responses described here were chosen for their relevance to other high-pressure phenomena. For example, because *S. cerevisiae* exhibits an adaptive response to pressurization [44], we hypothesized that pressure cycling might lead to longer-term changes to the metabolism. The observation of a cycle-dependent autofluorescence response (Figure 2) shows promise for this line of investigation. Next, because pressure affects fermentation [18], we decided to investigate the autofluorescence response to ethanol. The observation of a pressure-dependent autofluorescence response to ethanol (Figure 5 and Figure 6) suggests the potential for future work in this area as well.

By demonstrating how NADH and NADPH conformation can serve as a real-time metabolic indicator under pressurized conditions, we expand the use of hydrostatic pressure as a biophysical tool. Pressure or pressure jumps might be useful for the non-thermal activation or inhibition of metabolic processes or for modulating the coupling between processes. For these reasons, exploring metabolism under extreme physiological conditions may reveal new biology.

## 4. Materials and Methods

### 4.1. Instrumentation

The spectrofluorometric system was described previously [32] and consisted of a nitrogen-gas discharge laser (model GL-3300, Photon Technology International, Birmingham, NJ, USA; 337 nm wavelength, 1 ns nominal pulse width) as the excitation source with excited-state emission acquired using a spectrograph (model MS125, Newport, Irvine, CA, USA) coupled to a nanosecond-gated intensified CCD (ICCD) (model iStar734, Andor, Belfast, UK). The ICCD gate was timed to open 5 ns prior to the arrival of emission and the gate width was set to 80 ns, which is sufficient for acquiring the entire time-integrated emission signal. Measured spectra were dark-current corrected and consisted of 1024 spectral channels covering an interval between 400 and 650 nm wavelength.

The sample was housed in a custom-built high-pressure microscopy imaging chamber [48] used as a spectroscopic cell. The chamber consisted of a 1.5 × 0.5 mm outer-to-inner diameter quartz capillary (Q150-50-7.5, Sutter Instrument, Novato, CA, USA) epoxy-sealed to commercially available high-pressure, stainless-steel tubing (High Pressure Equipment Company, Erie, PA, USA).

For solution measurements, the chamber was attached to a high-pressure system consisting of a manually cranked positive-displacement pressure generators (37-63-0, High Pressure Equipment Company), pressurizing-medium reservoir, pressure gauge, and valves. The pressurizing medium was a 50/50 ethanol/water mixture. Contact between the pressurizing medium and sample was made approximately 1 m upstream of the probe region so that any mixing between the sample and pressurizing medium would not be detected. Pressure was measured using a Bourdon strain gauge (6PG30, High Pressure Equipment Company).

For cellular measurements, the chamber was attached to a microperfusion system [49], consisting of two positive-displacement pressure generators (same model as above) where one generator was advanced to create flow while a second generator was retracted at a rate based on a measurement of pressure from a digital manometer (LEX1, Keller AG, Winterthur, Switzerland). Generators were driven by a stepper motor coupled to a high-torque gearhead; stepper motors were controlled via LabVIEW-based computer interface. Manual high-pressure valves were used to switch between two solution reservoirs, allowing for the chemical environment of the cellular sample to be changed while pressurized.

### 4.2. Sample Preparation and Data Acquisition

For NADH solutions containing methanol, a 10× concentrated NADH stock (400 µM NADH (cat. no. N8129, Sigma-Aldrich, St. Louis, MO, USA), 200 mM MOPS, pH 7.4), spectroscopic-grade methanol (cat. no. 154903, Sigma-Aldrich), and deionized water were mixed in either 1:0:9 or 1:9:0 ratios for final concentrations of 40 µM NADH, 20 mM MOPS, and 0 or 90 vol% methanol.

For NADH solutions containing malate dehydrogenase (MDH), 5 µM NADH was prepared in 20 mM MOPS buffer, pH 7.4. An ammonium–sulfate precipitate of MDH from porcine heart (cat. no. M1567, Sigma-Aldrich) was added to the NADH solution without additional purification. Protein concentration was calculated assuming a homodimer molecular weight of 70 kDa [50] and using the manufacturer’s lot analysis for protein content. The added volume was accounted for when calculating final protein and NADH concentrations. A binding constant *K* = (2.6 ± 0.1) × 10^5^ M^–1^ [51] was used for estimating the fraction of NADH bound to MDH. MDH has two independent and identical NADH binding sites per protein homodimer [51], and so the binding site concentration was twice the protein concentration. 

NADH and NADH/protein solutions were loaded into the capillary chamber by flushing several chamber volumes worth of sample before sealing at one end using a standard cone seal and attaching the other end to the pressure generator. NADH solutions were used within a day of preparation, and proteins were kept refrigerated until just prior to use. Samples were not temperature regulated; room temperature was measured at 22 ± 2 °C.

Spectral measurements were performed at pressures of 0.1 MPa (ambient pressure) to 41.4 MPa (6 kpsi) in 10.3 MPa (1.5 kpsi) increments with the sample equilibrated at a given pressure for more than 5 min before spectral acquisition. To confirm there was negligible photobleaching, measurements were made at each pressure during both pressurization and depressurization of the sample. For another measurement, pressure was cycled between 1.38 MPa (0.2 kpsi) and 33.1 MPa (4.8 kpsi) with the sample equilibrated at a given pressure for more than 5 min before spectral acquisition. To confirm there was a negligible effect due to photobleaching, measurements using multiple excitation intensities were compared.

For cellular measurements, *S. cerevisiae* was grown on YPD agar medium (cat. no. Y1000, TekNova, Hollister, CA, USA) for two or three days rather than in liquid medium to minimize background fluorescence. Prior to measurement, cells were triple washed in phosphate-buffered saline (PBS, cat. no. 20012, Life Technologies, Carlsbad, CA, USA) before being suspended in PBS. Samples prepared in this manner were confirmed to have a UV-excited autofluorescence intensity that responded to oxygenation and to additions of cyanide, ethanol, and glucose [31] in a manner similar to starved yeast cultures maintained in a batch reactor at ambient pressure [41].

The protocol for acquiring autofluorescence data from cellular samples housed in a UV-transparent spectroscopic cuvette is described previously [33].

To acquire autofluorescence data from cellular samples under pressure, the capillary chamber was modified to immobilize non-adherent cells by creating a partial epoxy plug that trapped a smaller inner quartz capillary (flame heated and pulled to be less than 0.5 mm OD, then flame sealed on one end). Cellular suspensions were added to the inner capillary using a syringe; then, they were lightly centrifuged (a few seconds in a tabletop centrifuge). The capillary was cut to size (1.5 cm in length) and manually flowed into the chamber with the sealed end pointing downstream until it reached the epoxy plug. After visual confirmation of sample immobilization, the capillary chamber was attached to the microperfusion system. Since the epoxy plug did not fully occlude the chamber, the perfusion medium flowed around the inner capillary. The upstream end was not sealed, allowing the perfusion medium to exchange with the suspension medium.

To chemically induce a metabolic response, a valve to the first reservoir was closed while a valve to the second reservoir was simultaneously opened. For this study, the initial reservoir contained PBS only and the second reservoir contained 1 µM rhodamine B in PBS and either cyanide or ethanol at the concentration indicated. A higher concentration was used than for cuvette-based measurements to account for any mixing of solutions between reservoirs. Rhodamine B was used as an indicator for the arrival of cyanide or ethanol at the sample. Cyanide (cat. no. 60178, Sigma-Aldrich) is a respiratory inhibitor that binds to Complex IV of the electron transport chain [40]. Ethanol (cat. no. 459828, Sigma-Aldrich) is believed to have effects at multiple points [38,39,52]. Cellular samples were not temperature regulated; room temperature was measured at 22 ± 2 °C.

To confirm that changes in autofluorescence were not due to artifacts including photobleaching, insufficient perfusion, and sample loss, autofluorescence was monitored at a fixed pressure without inducing a chemical response using a range of excitation intensities and perfusion rates. For the pressures reported here (up to 32 MPa), the rate of emission intensity decrease was less than 0.5%/min below an energy of 10 µJ/pulse excitation at the sample and did not appear to be reduced by further attenuating the excitation. Next, the 0.5%/min rate of emission intensity decrease was insensitive to perfusion rates ranging from 1 to 3 µL/s, suggesting that conditions were not in a regime limited by perfusion rate. For comparison, the probe-region volume is estimated to be at most 20 µL, which is based on the apparent size of the excitation region. An energy of 6 µJ/pulse and perfusion rate of 1 µL/s were used for cellular results presented here. Occasionally, larger intensity loss rates were observed even under these conditions. Given that the optics were aligned so that excitation occurred near the open end of the inner capillary, we attributed these larger rates of intensity loss to sample loss, i.e., the non-adherent cells being carried away by perfusion.

For results presented here, three criteria needed to be satisfied for an autofluorescence response to be deemed chemically induced: (1) the increase in rhodamine emission had to indicate an unambiguous arrival time for the chemical, (2) the rate of autofluorescence decrease had to be small prior to chemical arrival, and (3) the average autofluorescence wavelength prior to chemical arrival had to be steady with any change in behavior corresponding unambiguously to the arrival of the rhodamine signal.

### 4.3. Spectral Phasor Analysis

Initially developed for the rapid identification of regions within hyperspectral images [53], spectral phasor analysis as used here is described in related publications [31,33,34]. Briefly, a spectral phasor is

(1) $$A=\underset{j}{\sum }F_{j}e^{i\frac{2\pi }{N}j}$$

where *F* is the spectrum normalized to the integrated intensity, *j* is the spectral channel, and *N* is the number of spectral channels. A phasor can be mapped onto a two-dimensional plot of its real and imaginary components,

(2) $$\begin{array}{c} Re\left(A\right)=\underset{j}{\sum }F_{j}\cos\left(\frac{2\pi }{N}j\right)\\ Im\left(A\right)=\underset{j}{\sum }F_{j}\sin\left(\frac{2\pi }{N}j\right) \end{array}.$$

For a spectral interval (i.e., a set of spectral channels) that is centered on a Gaussian-shaped spectrum, *Re*(*A*) < 0 and *Im*(*A*) = 0. If the spectrum exhibits a small shift to longer wavelength, *Im*(*A*) will decrease in value and vice versa. If the spectrum instead becomes narrower in width, *Re*(*A*) → –1, and if the spectrum becomes wider in width, *Re*(*A*) → 0. Figures mapping out and further explaining this behavior are found elsewhere [34,53]. Spectral intervals for phasor calculations (specified in the figure captions) are chosen so that the measured spectrum is roughly centered in the interval.

Note that for spectral change acting as a superposition of two spectra, phasors are collinear when plotted *Re*(*A*) *versus Im*(*A*). To show this, assume a measured, normalized spectrum *F* to be a linear combination of two spectra *F*_1_ and *F*_2_, which is weighted by a fraction *a* ranging from 0 to 1. Then, the spectral phasor is

(3) $$A=\underset{j}{\sum }F_{j}e^{i\frac{2\pi }{N}j}=\underset{j}{\sum }\left[aF_{1,j}+\left(1-a\right)F_{2,j}\right]e^{i\frac{2\pi }{N}j}=aA_{1}+\left(1-a\right)A_{2}\;$$

which forms a line on a *Re*(*A*)-*versus*-*Im*(*A*) plot as *a* is varied because phasors behave graphically as a vector. Solving for *a*, we have

(4) $$a=\frac{\left(A-A_{2}\right)}{\left(A_{1}-A_{2}\right)}\;.$$

Thus, *a* is the fractional distance between the phasors *A*_2_ and *A*_1_.

In practice, phasor values calculated from the autofluorescence of independently prepared samples show small variation due to differences in the optical path, variability between samples, etc. When assessing the collinearity of phasor responses across multiple samples, it is helpful to shift the axes so that the time-averaged initial phasor is at the origin. Such phasor plots will have axes labeled Δ*Re*(*A*) and Δ*Im*(*A*) as opposed to *Re*(*A*) and *Im*(*A*). In theory, the direction of a phasor response depends on this variation. Nonetheless, if the sample-to-sample variation is small, the effect on phasor-shift direction is not significant enough to affect collinearity [34].

Spectral phasor analysis has been used to sense reduced pyridine nucleotide conformation at ambient pressure in cellular systems [31,32] with a metabolic interpretation for chemically induced autofluorescence response being developed [33,34]. Specifically, we show that two-component spectral behavior occurs when metabolic change involves the same response mechanism, and non-two-component behavior is possible when metabolic change involves different response mechanisms [33]. For example, sequential chemical additions of ethanol and cyanide into a sample gave non-two-component responses while sequential additions of d-glucose and deoxyglucose gave two-component responses. Further, l-glucose (a metabolically inactive enantiomer) gave no response with subsequent d-glucose and deoxyglucose additions once again giving a two-component response. Other controls demonstrated how the autofluorescence response was not an artifact of the chemical addition; e.g., a given chemical (i.e., cyanide) gave different, non-collinear responses depending on sample incubation in glucose and, conversely, different chemicals (i.e., various alcohols) gave collinear responses of varying magnitude correlating to the degree of respiratory inhibition by each alcohol.

Applications using phasor analysis to assess two-component behavior in the autofluorescence emission response include distinguishing respiratory and oxidative stress responses associated with NADH and NADPH despite their near-identical emission properties [34] and demonstrating the detection of a metabolic response from cells embedded in tissue-like environments containing strong, spectrally similar background emission [54].

## 5. Conclusions

The observations of UV-excited cellular autofluorescence response presented here point to new avenues of investigation in piezophysiology. First, a comparison between the pressure-induced autofluorescence response with emission properties of NADH suggest a pressure-induced change in redox state and ROS production. Next, although two respiratory inhibitors (cyanide and ethanol) elicit similar autofluorescence responses at ambient pressure, the ethanol-induced response is altered under pressurized conditions, which is consistent with broader observations that membrane-based processes exhibit pressure sensitivity. Overall, spectral phasor analysis helps to identify two-component spectral behavior in an autofluorescence response to assess the involvement of either one or multiple NADH- and NADPH-linked pathways. Spectral phasors provide a way to visualize energy metabolism and mitochondrial function under pressurized conditions by revealing pressure-dependent dynamics in these metabolisms.

## Data Availability

Data are available upon reasonable request.

## Sample Availability

Samples of the compounds are not available from the authors.
